# Proof–of–concept evidence for high–density EEG investigation of sleep slow wave traveling in First-Episode Psychosis

**DOI:** 10.1038/s41598-024-57476-2

**Published:** 2024-03-21

**Authors:** Anna Castelnovo, Cecilia Casetta, Simone Cavallotti, Matteo Marcatili, Lorenzo Del Fabro, Maria Paola Canevini, Simone Sarasso, Armando D’Agostino

**Affiliations:** 1 Sleep Medicine Unit, Neurocenter of Italian Switzerland, Ente Ospedaliero Cantonale (EOC), Via Tesserete 46, 6900 Lugano, Switzerland; 2 Faculty of Biomedical Sciences, University of Italian Switzerland, Lugano, Switzerland; 3 University Hospital of Psychiatry and Psychotherapy, University of Bern, Bern, Switzerland; 4 Department of Mental Health and Addiction, ASST Santi Paolo e Carlo, Via A. Di Rudinì 8, 20142 Milan, Italy; 5 Institute of Psychiatry, Psychology and Neuroscience, King’s College London, London, UK; 6 Psychiatric Department, ASST Monza, San Gerardo Hospital, Monza, Italy; 7 Department of Pathophysiology and Transplantation, Università degli Studi di Milano, Milan, Italy; 8 Department of Neurosciences and Mental Health, IRCCS Fondazione Ca’ Granda Ospedale Maggiore Policlinico, University of Milan, Milan, Italy; 9 Department of Health Sciences, Università degli Studi di Milano, Milan, Italy; 10 Department of Biomedical and Clinical Sciences “L. Sacco”, Università degli Studi di Milano, Via G.B. Grassi 74, 20157 Milan, Italy

**Keywords:** Connectivity, Brain plasticity, Schizophrenia, Early course psychosis, Slow waves, Clozapine, Diagnostic markers, Slow-wave sleep

## Abstract

Schizophrenia is thought to reflect aberrant connectivity within cortico-cortical and reentrant thalamo-cortical loops, which physiologically integrate and coordinate the function of multiple cortical and subcortical structures. Despite extensive research, reliable biomarkers of such "dys-connectivity" remain to be identified at the onset of psychosis, and before exposure to antipsychotic drugs. Because slow waves travel across the brain during sleep, they represent an ideal paradigm to study pathological conditions affecting brain connectivity. Here, we provide proof–of–concept evidence for a novel approach to investigate slow wave traveling properties in First-Episode Psychosis (FEP) with high-density electroencephalography (EEG). Whole–night sleep recordings of 5 drug-naïve FEP and 5 age- and gender-matched healthy control subjects were obtained with a 256-channel EEG system. One patient was re-recorded after 6 months and 3 years of continuous clozapine treatment. Slow wave detection and traveling properties were obtained with an open-source toolbox. Slow wave density and slow wave traveled distance (measured as the line of longest displacement) were significantly lower in patients (*p* < 0.05). In the patient who was tested longitudinally during effective clozapine treatment, slow wave density normalized, while traveling distance only partially recovered. These preliminary findings suggest that slow wave traveling could be employed in larger samples to detect cortical "dys-connectivity" at psychosis onset.

## Introduction

Schizophrenia (SCZ) is a mental disorder characterized by perceptive (hallucinations), cognitive (disorganized speech and behavior, delusions) and even subtle motor abnormalities^1^. Ranked by the World Health Organization among the 10 leading causes of disability in adults, SCZ has an estimated world-wide prevalence up to 1%^2–4^.

Despite extensive research, the pathophysiology of this clinical syndrome remains elusive. Specific symptoms, like hallucinations, have been convincingly associated with specific brain structural and functional abnormalities^5–7^. However, the complex and heterogeneous symptomatology observed could not be localized to specific cortical regions^8^, nor attributed to abnormal activity of the brain cortex alone^9^. Hence, the search of a unifying physio-pathogenetic mechanism to explain the many available and often contradictory findings remains challenging^10^. The so-called “dysconnectivity hypothesis”^11^ offers a possible solution by pointing to a defective integration among distributed brain regions. According to this hypothesis, signs and symptoms of SCZ can be explained by the aberrant connectivity within cortico-cortical and reentrant thalamo-cortical loops, which physiologically integrate and coordinate the function of multiple cortical and subcortical structures. Based on Eugene Bleuler’s original conception of disturbed integration of higher cognitive functions as the core of SCZ, this hypothesis has now begun to receive partial confirmation from neuroimaging studies^12,13^. Although the full spectrum of thalamocortical connectivity abnormalities remains to be elucidated, recent evidence suggests reduced connectivity between prefrontal cortical structures and the thalamus may be detectable in the early stages of psychosis^14^.

Among the many techniques and methods available to study brain connectivity in SCZ, sleep electroencephalographic (EEG) recordings offer 4 major advantages. First, the unique possibility to minimize possible confounding factors related to waking activities (including changes in the level of attention, decreased motivation or cognitive capacity, and the presence of active symptoms). Second, the possibility to relate specific EEG patterns to specific neural circuits and functions^15^, thanks to the fast-growing advancements in the field of sleep neurophysiology. Thirdly, EEG, the preferred tool for sleep investigation, is a non-invasive and cost-effective technique that enables brain imaging without hindrance from normal or abnormal nocturnal movements. Its exquisite temporal resolution provides detailed insights throughout the entire sleep cycle. In addition, high-density EEG (hd-EEG)—derived from the recent implementation of traditional EEG systems—offers a reasonably high spatial resolution, thanks to an increased number of scalp electrodes (up to 256 sensors with 1 cm resolution). Fourth, recent reviews of several studies employing EEG in SCZ confirmed the presence of abnormalities in sleep oscillations in affected patients^16–18^.

Specifically, sleep slow waves, the hallmark of stage 3 non-rapid eye movement (NREM) sleep, were reported to be reduced in unmedicated patients with SCZ, even at early stages of the disease^19^. Slow waves are known to reflect the integrity of thalamocortical circuits^15,20^, and have been associated with brain plasticity, memory and more broadly to several cognitive functions^21^. EEG slow waves appear as continuous high amplitude (> 75 µV) slow oscillations in the 1–4 Hz range, underpinned by the alternation between neuronal hyperpolarization and silence lasting dozens or hundreds of milliseconds (corresponding to the wave down-state or negative peak), periodically interrupting neuronal tonic depolarization and firing (corresponding to the slow wave up-state). These cellular phenomena occur quasi-synchronously across large cortical sections, so that they can be recorded using scalp EEG. Slow waves are generated and spread by the cortex^22^, although the thalami^23^ seem to play a fundamental role for their full expression^24–26^. More recently, subcortical white matter tracts^27^ were shown to sustain the so-called traveling^28^ of larger slow waves, which typically originate from a definite site and travel over the scalp at an estimated speed of 1.2–7.0 m/sec. The pattern of origin and propagation of sleep slow oscillations is reproducible across nights and subjects and provides a blueprint of cortical excitability and connectivity. We recently showed that slow wave traveling is a viable method to study connectivity in SCZ first-degree relatives (FDRs)^29^ and that slow wave properties are a candidate endophenotype for SCZ^30^.

Here we propose slow wave traveling as a candidate marker of aberrant cortico–cortical/thalamocortical connectivity at the onset of SCZ and we report a single case study and an explorative case–control analysis on a small sample of patients as proof–of–concept.

## Methods

### Participants

All patients who were hospitalized for First-Episode Psychosis (FEP) in the psychiatric ward of the San Paolo University Hospital over a period of 1 year were asked to participate in a larger study on sleep EEG in patients, unaffected first–degree relatives and healthy control subjects approved by the Milan Area 1 ethics committee. The study conformed with World Medical Association Declaration of Helsinki published on the website of the Journal of American Medical Association in 2013. One patient agreed to two follow-up sleep recording sessions which were performed at 6 months and 3 years after hospitalization, respectively. Informed consent was obtained from all the study participants. During the study period, only 5 Caucasian drug-naïve patients were recorded with a 256–channel EEG system (Electrical Geodesics Inc., Eugene, OR). Patients (n = 5, 100% males, 24.4 ± 3.58 years old) were age– and gender–matched with healthy control individuals (n = 5, 100% males, 24.0 ± 3.32 years old) chosen among good sleepers with a negative history of previous or current medical, neurological or psychiatric diagnoses. Control subjects were also excluded if they had any FDR with a history of major psychiatric disorders.During their first hospitalization, 2 patients received a diagnosis of Schizophreniform Disorder whereas the others satisfied criteria for Psychotic Disorder Not Otherwise Specified (NOS) according to DSM-5^31^. The diagnosis of SCZ was confirmed for all subjects after a 2-year follow-up period by at least 2 expert clinicians through direct interview and medical charts review. All participants were antipsychotic-naïve and free from any other medication that could affect sleep for a minimum of 1 month at the time of recording. Estimated mean durations of untreated illness and psychosis at the time of hospitalization were respectively 13.17 ± 9.87 months and 7.17 ± 2.90 weeks.

### Clinical characterization

Major sleep disorders were screened through the participant’s clinical history and the Pittsburgh Sleep Quality Index (PSQI), the Epworth Sleepiness Scale (ESS) and the STOP-bang Apnea Questionnaire (Table 1). At the PSQI, none reported snoring, pauses between breaths, leg twitching or jerking, episodes of disorientation and confusion, or restlessness while asleep. However, all reported sleep-onset or maintenance insomnia (more than 3 times per week), 3 reported bad dreams, and 4 reported of feeling too hot or too cold. Psychopathology was assessed using the Positive and Negative Syndrome Scale (PANSS)^32^, the 21-item Italian version^33^, and the Launay–Slade Hallucination Scale (LSHS-R)^34^, while cognition was assessed using the Brief Assessment of Cognition in Schizophrenia (BACS)^35^.

**Table 1:** FEP clinical features.

ID	PSQI	SBAQ	ESS	PDI	LSHS-R	PANSS	BACS memory	BACS digit	bacs token	BACS symbol	BACS fluency	BACS London
PT1	5	1	7	6	7	95	32.25	13.75	61	18.25	38.25	10.75
PT2	6	1	0	13	30	85	41	20.75	62	38	31.5	11
PT3	7	1	8	10	25	84	28.25	17.75	47	35.25	29.25	14.75
PT4	4	1	2	11	13	61	42.25	21.5	73.25	47.5	73.25	18.75
PT5 T0	11	1	2	17	34	110	42	23.75	38.25	31.25	57.25	15.75
PT5 T1	–	–	–	–	–	58	59.25	24.75	65	35.25	54.25	12.75
PT5 T2	–	–	–	–	–	45	55.25	27.75	77	40.25	55.25	14.75

Rows: PT 1–5: patients numbered from 1 to 5. PT5 T0: patient number 5 at baseline, drug-naïve (acute psychotic symptoms before the start of treatment). PT5 T1: patient number 5 after 6 months of antipsychotic treatment, clozapine 200 mg/day at the time of the recording. PT5 T2: patient number 5 after 3 years of treatment, clozapine 75 mg/day at the time of the recording.

Columns: PSQI: Pittsburgh Sleep Quality Index. SBAQ: Stop Bang Apnea Questionnaire. ESS: Epworth Sleepiness Scale. PDI: Peters et al. Delusion Inventory. LSHS-R: Launay–Slade Hallucination Scale. PANSS: Positive And Negative Syndrome Scale. BACS: Brief Assessment of Cognition in Schizophrenia (corrected scores). Memory: Verbal Memory & Learning (Verbal Memory). Digit: Working Memory (Digit Sequencing). Token: Motor Function (Token Motor Task). Symbol = Speed of Processing (Symbol Coding). Fluency: Verbal Fluency (Semantic and Letter Fluency). Tower = Executive Function (Tower of London).

FEP clinical features are summarized in Table 1.

### Sleep EEG analysis

All subjects underwent an overnight hd-EEG recording (256 channels; Electrical Geodesics Inc., Eugene, OR, 500 Hz, vertex-reference). One of the 5 patients (PT5), whose clinical history has been detailed elsewhere^36^, was reassessed at different time-points (after 6 months and 3 years):

- At T0 or baseline, before starting an antipsychotic treatment.
- At T1, after 6 months of pharmacotherapy (3 months of titration and months of clozapine 200 mg per day)
- At T2, after 3 years (last 2 of clozapine at the maintenance dosage of 75 mg per day).

For all EEG recordings, lights-out fell within 1 h of the participants most consistently reported bedtime, while light-on varied as subjects were allowed to sleep ad libitum. All EEG signals were imported in MATLAB (The MathWorks Inc., Natick, MA) and high-pass filtered at 0.1 Hz, down-sampled to 128 Hz, band-pass filtered (2-way least-squares FIR, 1–40 Hz). Sleep staging was performed in Matlab, with the support of a user graphical interface (https://github.com/Mensen/swa-matlab), according to standard criteria^37^ by a physician certified in Sleep Medicine (AC). Submental electromyogram (EMG) and electro-oculograms (EOGs) were selected from channels around the neck, jaw and eyes. Semiautomatic artifact rejection procedures were utilized to remove channels and epochs with high frequency noise or interrupted contact with the scalp, as done in other recent studies^29,30,38^. Channels on the neck or face were removed, yielding 185 channels overlaying the scalp.

Spectral analysis in the slow wave activity range (SWA, 1–4 Hz) was performed using all clean 6-s epochs within NREM sleep (Welch’s averaged modified periodogram with a Hamming window) on average-referenced EEG signal. Sleep slow wave detection was conducted on mastoid-referenced signal using an open-source Matlab-based toolbox^39^. Parameters of interests were: slow wave density (count of slow waves per minute), negative peak amplitude, travelled distance (measured as the line of longest displacement).

### Statistical analysis

Between-group statistical comparisons of demographic and sleep architecture variables were performed using unpaired 2-tailed t-tests, Mann–Whitney U tests, or χ^2^ tests, as appropriate. Normality of data and homogeneity of variance were evaluated using the Shapiro/Wilk’s test and Levene’s test, respectively. For slow wave topographical analysis, we applied non-parametric statistical mapping using a non-parametric suprathreshold cluster analysis to control for multiple comparisons^40^, as previously described^38,41^.

## Results

### Sleep architecture

FEP patients showed a trend for an increase in wake after sleep onset and NREM stage 1 (%), and for a decrease in NREM stage 3 and sleep efficiency (Table 2).

**Table 2:** Sleep Architecture group-comparison.

ID	Control	FEP	*p*
TIB	426.6 ± 48.86	458.2 ± 76.32	0.46
TST	384.8 ± 43.24	332.9 ± 126.93	0.43
WASO	34.7 ± 19.49	113.6 ± 65.37	0.05*
SE	90.26 ± 3.93	70.79 ± 17.83	0.07
N1	20.26 ± 7.97	31.7 ± 16.51	0.21
N1%	5.29 ± 2.02	9.35 ± 3.59	0.07
N2	211.5 ± 27.17	206.3 ± 115.96	0.93
N2%	54.92 ± 2.51	58.74 ± 12.77	0.55
N3	70.8 ± 11.56	38.4 ± 32.85	0.09
N3%	18.68 ± 3.96	14.06 ± 15.79	0.56
REM	82.3 ± 22.54	56.5 ± 25.24	0.13
REM %	21.13 ± 3.84	17.86 ± 8.36	0.46
REML	85.7 ± 26.12	170.6 ± 126.52	0.21
AI	8.35 ± 2.97	9.38 ± 3.02	0.6

Control: good sleeper healthy controls used for the case series. FEP: First-Episode Psychosis patients used for the case series. TIB: time in bed (minutes). TST: total sleep time (minutes). WASO: wake after sleep onset (minutes). SE: modified sleep efficiency (percentage) = total recording time % /TST. N1: minutes spent N1. N1%: TST spent in N1 (%). N2: minutes spent N2. N2%: TST spent in N2 (%). N2: minutes spent N2. N3%: TST spent in N3 (%). REM %: TST spent in REM (%). REML: REM latency. AI: arousal index; * significant difference between groups.

**Figure 1:**
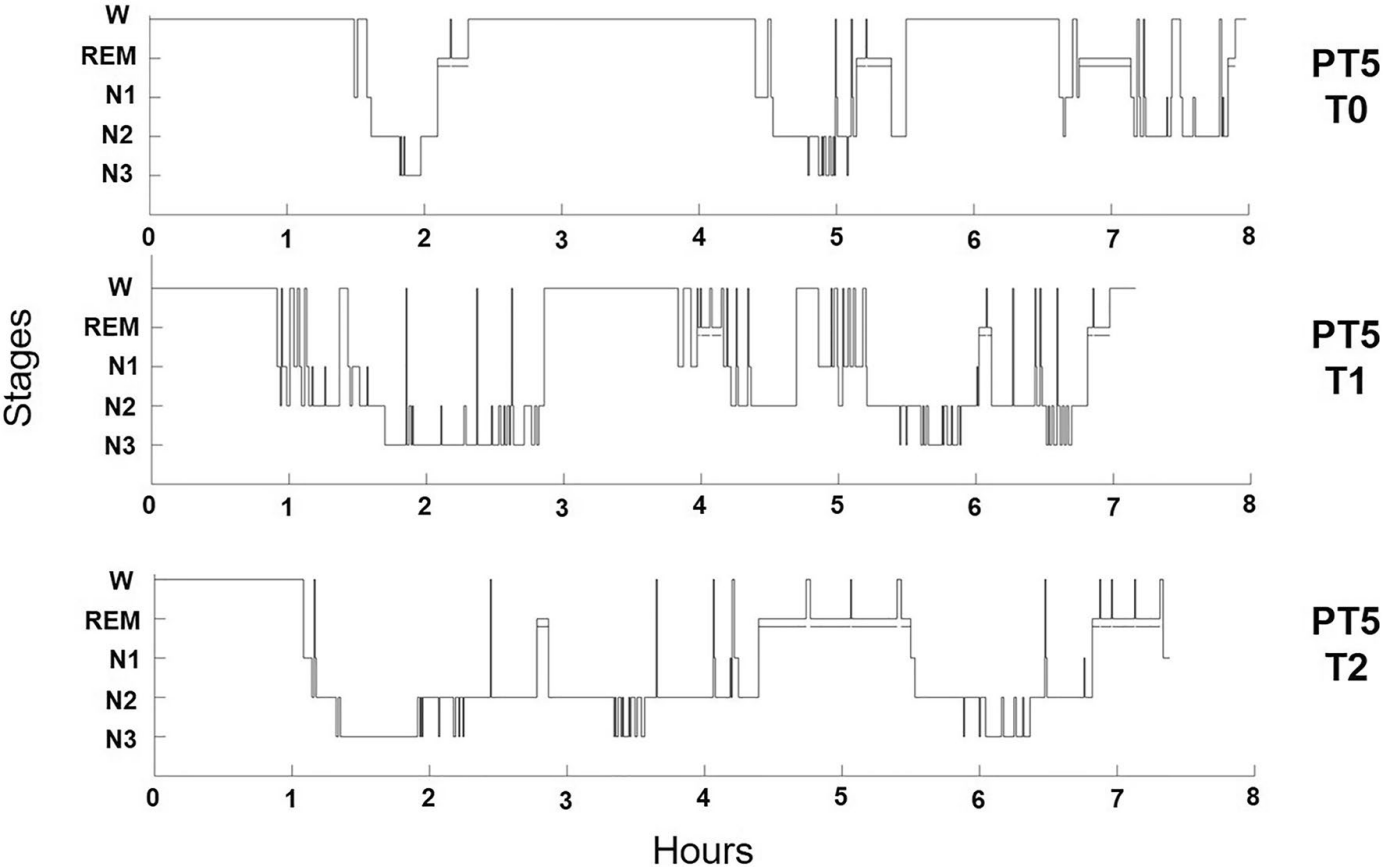
Sleep Architecture of the one patient tested longitudinally (PT5). W = Wake. REM: Rapid Eye Movement Sleep. N1: non REM stage 1. N2: non REM stage 2. N3: non REM stage 3. PT5 T0 = patient 5 at baseline, drug-naïve (acute psychotic symptoms before starting treatment). PT5 T1 = patient 5 after 6 months of antipsychotic treatment, clozapine 200 mg/day at the time of the recording. PT5 T2 = patient 5 after 3 years of treatment, clozapine 75 mg/day at the time of the recording.

PT5 slept progressively better from T0 to T2 (Fig. 1), with an improvement in SE, and a reduction in WASO, to a level comparable to matched control at T2. Reciprocally, REML, N3 and N3% were markedly reduced at T0, increased at T1 (while the opposite is observable for REM and REM %), and appeared normalized at T2 (Table 3). N2 and N2% remained fairly stable across conditions in PT5, and qualitatively comparable to matched control subjects (Table 3).

**Table 3:** Sleep Architecture of the one patient tested longitudinally (PT5).

ID	TIB	TST	WASO	SE	N1	N1%	N2	N2%	N3	N3%	REM	REM %	REML	AI
PT5 T0	417.5	188.5	220.5	45.2	24	12.73	99	52.52	9.5	5.0	56	29.71	39.5	7.6
PT5 T1	394.5	286.5	98.0	72.6	38.0	13.3	144.5	50.4	79.5	27.7	24.5	8.6	199.5	9.4
PT5 T2	405.5	391.5	9.0	96.5	13.6	3.5	212.5	54.3	67.5	17.2	101.0	25.8	110.5	7.5

TIB: time in bed (minutes). TST: total sleep time (minutes). WASO: wake after sleep onset (minutes). SE: modified sleep efficiency (percentage) = total recording time % /TST. N1: minutes spent N1. N1%: TST spent in N1 (%). N2: minutes spent N2. N2%: TST spent in N2 (%). N2: minutes spent N2. N3%: TST spent in N3 (%). REM %: TST spent in REM (%). REML: REM latency. AI: arousal index.

PT5 T0: patient 5 at baseline, drug-naïve (acute psychotic symptoms before starting treatment). PT5 T1: patient 5 after 6 months of antipsychotic treatment, clozapine 200 mg/day at the time of the recording. PT5 T2: patient 5 after 3 years of treatment, clozapine 75 mg/day at the time of the recording.

### Power

**Figure 2:**
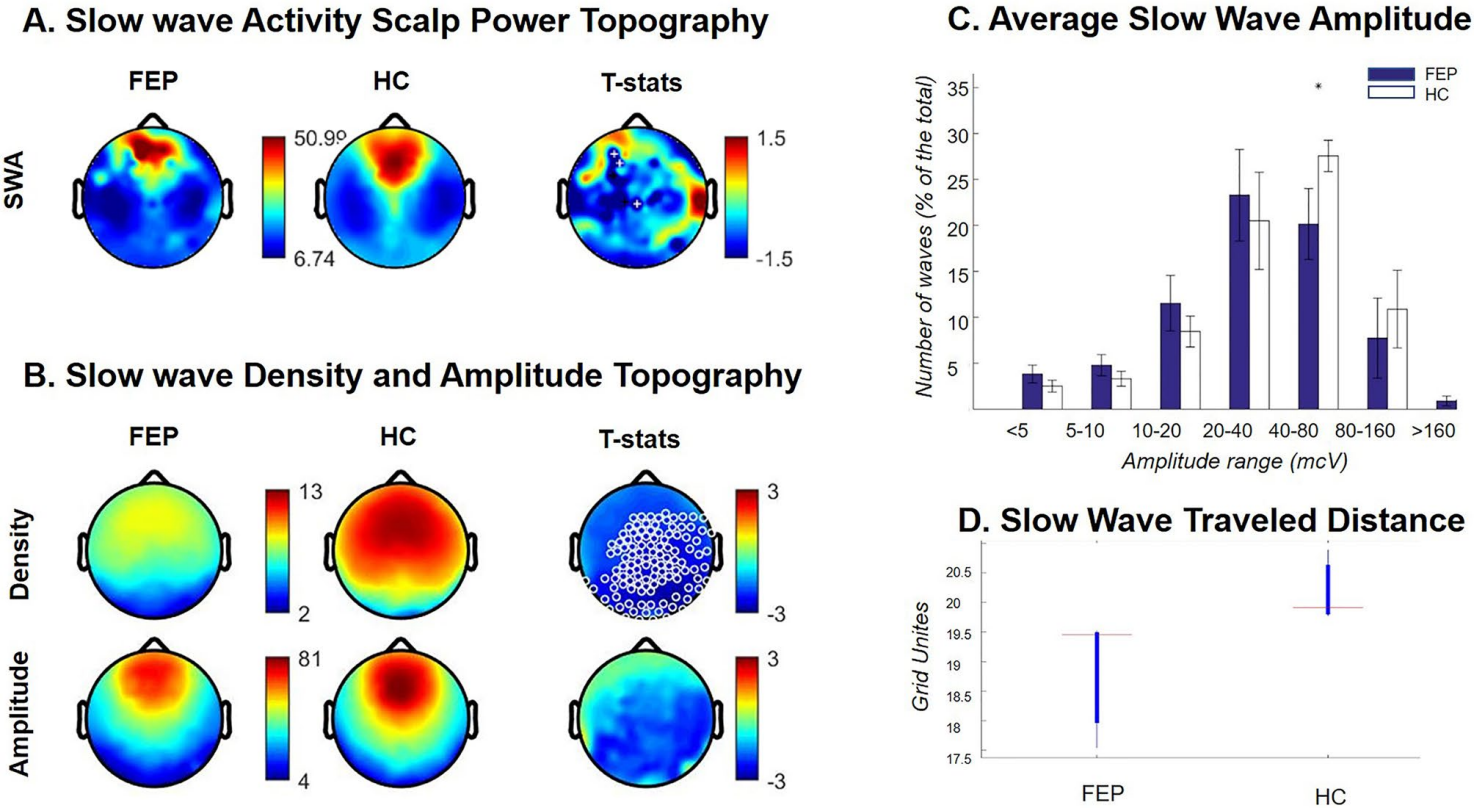
Case Series Analysis. (Panel **A**) SWA power topographical comparison. First column: average NREM sleep power EEG topographies in the FEP group. Second column: average NREM sleep power EEG topographies in the HC group. Third column: Map showing the individual electrode t-value (two-tailed, unpaired) maps for the comparison between FEP and HC in terms of absolute power. Fourth column: Same as third column except that each subject was spatially normalized using the z-score across electrodes before creating the t-value comparison. White crosses indicate individual electrodes that reached statistical significance (2 tailed, unpaired t-test, *P* ≤ 0.05). BLUE: patient < control, RED: patient > control. Channels on the neck or face were removed, yielding 185 channels overlaying the scalp. (Panel **B**) Topographical maps of slow wave parameters. First row: Slow wave density (number per hour of N2N3 sleep) at each channel. Second row: slow wave mean-amplitude (average of negative peaks values for each channel). First column: FEP. Second column: HC. White circles indicate electrodes that reached statistical significance after correction for multiple comparison (*P* ≤ 0.05). BLUE: patient < control, RED: patient > control. (Panel **C**) Distribution of the amplitude of slow waves for FEP and controls. The number of waves was computed for groups of waves with increasing amplitude. Amplitude is expressed as the average across channels and across participants. Mean values (± standard error) are plotted as a percentage of the total number of waves. The asterisk indicates significance *P* < 0.05, single threshold corrected. (Panel **D**) Slow wave travelled distance. Slow wave traveled distance was calculated as the line of longest displacement. Units of measurement are referred to a grid of 40*40 tiles. First bar: PT: first-episode psychosis patients. Second bar: HC: good sleeper healthy control. FEP: First Episode Psychosis. HC: Healthy Controls.

Topographical maps revealed a non-significant (*p* > 0.05) reduction in SWA power in FEP compared to control group (Fig. 2, panel A).

**Figure 3:**
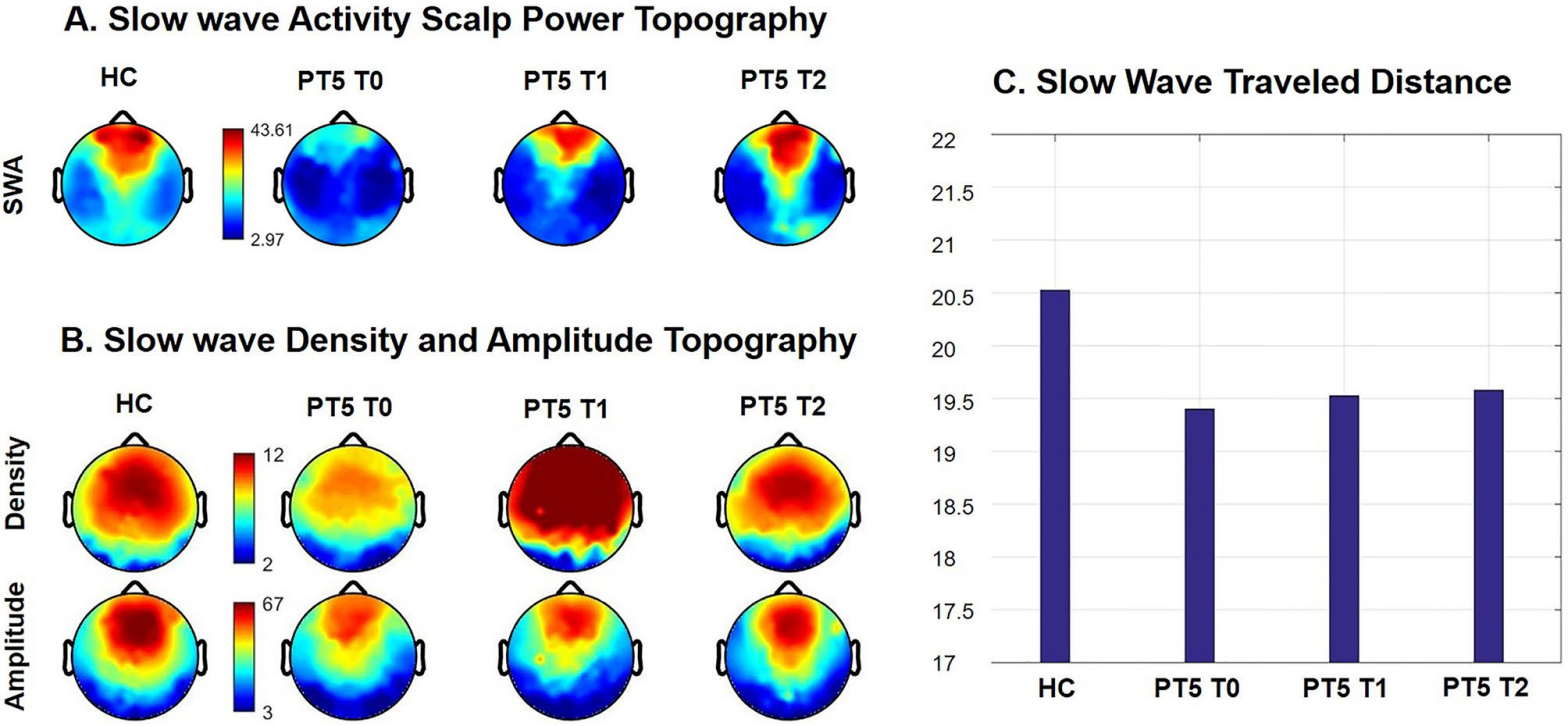
Longitudinal case report. (Panel **A**) SWA power topographical comparison between PT5 at different time-points and matched control Columns: average NREM sleep power EEG topographies. First column: HC. Second column: PT5 T0. Third column: PT5 T1. Fourth column: PT5 T2. BLUE: patient < control, RED: patient > control. Channels on the neck or face were removed, yielding 185 channels overlaying the scalp. (Panel **B**) Topographical maps of slow wave parameters. First row: Slow wave density (number per hour of N2N3 sleep) at each channel. Second row: slow wave average amplitude (average of negative peaks values for each channel). First column: HC. Second column: PT5 T0. Third column: PT5 T1. Fourth column: PT5 T2. BLUE: patient < control. RED: patient > control. (Panel **C**) Slow wave traveled distance. Distance was calculated as the line of longest displacement. Units of measurement are referred to a grid of 40*40 tiles. First bar: HC. Second bar: PT5 T0. Third bar: PT5 T1. Fourth bar: PT5 T2. HC = good sleeper healthy control. PT5 T0 = patient 5 at baseline, drug-naïve (acute psychotic symptoms before starting treatment). PT5 T1 = patient 5 after 6 months of antipsychotic treatment, Clozapine 200 mg/day at the time of the recording. PT5 T2: patient 5 after 3 years of treatment, Clozapine 75 mg/day at the time of the recording.

The qualitative observation of topographical maps (Fig. 3, panel A), suggest lower SWA in PT5 at T0 compared to matched control, and the subsequent progressive normalization of SWA from T1 to T2. *ï*

### Slow wave analysis

Slow wave analysis showed lower slow wave density at baseline in FEP versus control subjects (Fig. 2, panel B). This result remained significant after correction for multiple comparisons. A similar trend was observed for slow wave amplitude (Fig. 2, panel B and C), although group difference was not statistically significant at the topographical level after correction for multiple comparison (Fig. 2, panel B). However, when observing channel average amplitude at different percentiles, we could detect a selective reduction of high amplitude slow waves (Fig. 2, panel C).

Average traveled distance was significantly decreased in FEP vs control participants (*p* < 0.05), with almost no overlap between groups (Fig. 2, panel D).

At baseline, slow wave density for PT5 was visually lower compared to healthy control subjects. There was a progressive normalization from baseline to T2. In particular, there was a sharp increase at T1 and a return to levels comparable to the control participants at T2 (Fig. 3, panel B).

Slow wave traveled distance for PT5 was lower compared to healthy control subjects. There was a progressive increase from baseline to T2, yet without a full normalization of this parameter at T2 compared to healthy subjects (Fig. 3, panel C).

## Discussion

As sleep slow waves travel across the brain, their traveling properties represent an ideal paradigm to study pathological conditions interfering with brain connectivity. Taking advantage of this physiological paradigm and of the high spatial and temporal resolution of hd-EEG, here we investigated slow wave traveling at SCZ onset.

We observed a reduction of average travelled distance in 5 young adults at their first psychotic outbreak compared to age– and gender–matched control subjects. The lack of overlap between the 2 groups, makes the average traveled distance a putative candidate marker of SCZ to be tested in large, systematic studies. Of note, baseline traveled distance progressively increased but not normalized over time and during the course of effective treatment with clozapine in the only subject who was tested longitudinally. This temporal evolution seemed to parallel the progressive but partial recover of cognitive and social functions. It could be speculated that this incomplete recover reflects the underlying white matter damage in early course SCZ^42^. This interpretation is also consistent with the notion that long-range slow wave traveling is sustained by cortico-cortical connections^43–48^. Instead, slow wave density time-course recorded for this patient seemed to relate more closely to clozapine dosage. This preliminary data suggests that this latter observed abnormality could be related to active psychotic symptoms, and be partially reversible in early stages of SCZ.

Moreover, these preliminary data support the previous observation of an intrinsic deficit of slow wave generation in early course psychosis. Both density and amplitude appeared to be involved, with a significant shift in mean amplitude peak from 40–80 to 20–40 Hz. These observations are in line with the previous literature^16^. Lower slow wave number/density in NREM sleep in SCZ compared to matched control subjects was previously reported in 4 small-sized studies on drug-free and/or drug-naïve patients with SCZ^48–51^, and in one larger study on unmedicated patients with a diagnosis of SCZ Spectrum Disorder^52^. More recently, reduced slow wave power was also observed in a sample of 26 drug-naïve early course psychosis patients during NREM sleep stage 2^53^, and reduced slow wave density in a sample of 20 (medicated and unmedicated) early course psychosis patients during NREM sleep^19^. However, other studies failed to report differences in slow wave properties between patients with SCZ and control subjects^53–57^, perhaps due to the inclusion of many patients treated with Second-Generation Antipsychotics (SGA), which are known to affect slow wave power^16^. This interpretation is supported by the longitudinal observation of one subject, whose slow wave power and slow wave density appeared to closely reflect clozapine treatment. Although we cannot exclude this reflects the natural course of the patient’s disorder, the observed trend could point to a pharmacological effect driven by clozapine, which is known to increase global power, especially in lower frequency bands^58^. This interpretation may also explain why Ferrarelli et al.^19^ did not find differences in slow wave amplitude in their sample (almost half of the patients were taking SGA).

Last but not least, these findings were obtained using a standardized and open-source toolbox that can be readily employed in clinical contexts with expertise in sleep medicine and hd-EEG analysis. The use of comparable methodologies and tools across studies is critical to guarantee reproducibility of results, especially given the large heterogeneity observed in SCZ^29^.

The reported data is derived from a limited sample size comprising only 5 patients. Therefore, caution must be exercised in generalizing these findings to the broader patient population. Indeed, aberrant brain connectivity may vary across individuals, perhaps sustaining the well-known diversity in clinical subtypes and heterogeneous presentation of psychosis. Moreover, our sample is only representative of the male gender, and results could not be generalized to female patients, given the important role of gender in influencing sleep and brain EEG activity during sleep^59^. Nevertheless, our proof-of-concept study unequivocally demonstrates the feasibility of hd-EEG analyses in FEP. With its remarkable spatial and temporal resolution, this approach has the potential to unveil crucial insights into SCZ pathophysiology, offering valuable information for clinical, diagnostic, and prognostic purposes. Future studies should strive to collect larger clinical samples, confirm known sleep spindle abnormalities in FEP^60^, and clarify the relationship between spindle and slow wave activity.

## Conclusion

The preliminary findings outlined in this case report and case–control analysis: (1) support previous findings on slow wave deficits in the early stages of SCZ; (2) provide first evidence on slow wave traveling impairment at the onset of psychosis; (3) raise the question whether the early administration of clozapine may partially reverse slow wave abnormalities after the first psychotic episode; (4) offer a proof-of-concept regarding the potential utility of sleep slow wave traveling as a marker of brain dysconnectivity in SCZ; (5) confirm the utility of standardized algorithms and tools for sleep clinical studies; (6) encourage future systematic studies on larger cohorts of patients.

## Data Availability

Raw data are available upon request.

## References

[1] Poletti M, Gebhardt E, Kvande MN, Ford J, Raballo A (2019). Motor impairment and developmental psychotic risk: Connecting the dots and narrowing the pathophysiological gap. Schizophr. Bull..

[2] Knapp M, Mangalore R, Simon J (2004). The global costs of schizophrenia. Schizophr. Bull..

[3] McGrath J, Saha S, Chant D, Welham J (2008). Schizophrenia: A concise overview of incidence, prevalence, and mortality. Epidemiol. Rev..

[4] Simeone JC, Ward AJ, Rotella P, Collins J, Windisch R (2015). An evaluation of variation in published estimates of schizophrenia prevalence from 1990–2013: A systematic literature review. BMC Psychiatr..

[5] Hubl D (2004). Pathways that make voices: White matter changes in auditory hallucinations. Arch. Gen. Psychiatr..

[6] Hubl D (2010). Structural analysis of Heschl’s gyrus in schizophrenia patients with auditory hallucinations. Neuropsychobiology.

[7] Dierks T (1999). Activation of Heschl’s gyrus during auditory hallucinations. Neuron.

[8] Andreasen N (1986). Structural abnormalities in the frontal system in schizophrenia: A magnetic resonance imaging study. Arch. Gen. Psychiatr..

[9] Andreasen NC, Paradiso S, O’Leary DS (1998). “Cognitive dysmetria” as an integrative theory of schizophrenia: A dysfunction in cortical-subcortical-cerebellar circuitry?. Schizophr. Bull..

[10] Castelnovo A, Ferrarelli F, D’Agostino A (2015). Schizophrenia: From neurophysiological abnormalities to clinical symptoms. Front. Psychol..

[11] Friston KJ (1998). The disconnection hypothesis. Schizophr. Res..

[12] Canu E, Agosta F, Filippi M (2015). A selective review of structural connectivity abnormalities of schizophrenic patients at different stages of the disease. Schizophr. Res..

[13] Ramsay IS (2019). An activation likelihood estimate meta-analysis of thalamocortical dysconnectivity in psychosis. Biol. Psychiatr. Cogn. Neurosci. Neuroimaging.

[14] Sheffield JM (2020). Thalamocortical anatomical connectivity in schizophrenia and psychotic bipolar disorder. Schizophr. Bull..

[15] Adamantidis AR, Gutierrez Herrera C, Gent TC (2019). Oscillating circuitries in the sleeping brain. Nat. Rev. Neurosci..

[16] Castelnovo A, Graziano B, Ferrarelli F, D’Agostino A (2018). Sleep spindles and slow waves in schizophrenia and related disorders: Main findings, challenges and future perspectives. Eur. J. Neurosci..

[17] Ferrarelli F (2021). Sleep abnormalities in schizophrenia: State of the art and next steps. Am. J. Psychiatr..

[18] Lai M (2022). Investigating sleep spindle density and schizophrenia: A meta-analysis. Psychiatr. Res..

[19] Kaskie RE, Gill KM, Ferrarelli F (2019). Reduced frontal slow wave density during sleep in first-episode psychosis. Schizophr. Res..

[20] Steriade M (2003). The corticothalamic system in sleep. Front. Biosci..

[21] Huber R, Ghilardi MF, Massimini M, Tononi G (2004). Local sleep and learning. Nature.

[22] Beltramo R (2013). Layer-specific excitatory circuits differentially control recurrent network dynamics in the neocortex. Nat. Neurosci..

[23] Gent TC, Bandarabadi M, Herrera CG, Adamantidis AR (2018). Thalamic dual control of sleep and wakefulness. Nat. Neurosci..

[24] Zucca S, Pasquale V, de Leon Roig PL, Panzeri S, Fellin T (2019). Thalamic drive of cortical parvalbumin-positive interneurons during down states in anesthetized mice. Current Biol..

[25] Crunelli V, Hughes SW (2010). The slow (<1 Hz) rhythm of non-REM sleep: A dialogue between three cardinal oscillators. Nat. Neurosci..

[26] David F (2013). Essential thalamic contribution to slow waves of natural sleep. J. Neurosci..

[27] Avvenuti G (2020). Integrity of corpus callosum is essential for thecross-hemispheric propagation of sleep slow waves: A high-density EEG study in split-brain patients. J. Neurosci..

[28] Massimini M, Huber R, Ferrarelli F, Hill S, Tononi G (2004). The sleep slow oscillation as a traveling wave. J. Neurosci..

[29] Castelnovo A (2020). Slow wave oscillations in schizophrenia first-degree relatives: A confirmatory analysis and feasibility study on slow wave traveling. Schizophr. Res..

[30] D’Agostino A (2018). Sleep endophenotypes of schizophrenia: slow waves and sleep spindles in unaffected first-degree relatives. NPJ Schizophr..

[31] American Psychiatric Association (2013). Diagnostic and Statistical Manual of Mental Disorders.

[32] Kay SR, Fiszbein A, Opler LA (1987). The positive and negative syndrome scale (PANSS) for schizophrenia. Schizophr. Bull..

[33] Peters ER, Joseph SA, Garety PA (1999). Measurement of delusional ideation in the normal population: Introducing the PDI (Peters et al. Delusions Inventory). Schizophr. Bull..

[34] Serper M, Dill CA, Chang N, Kot T, Elliot J (2005). Factorial structure of the hallucinatory experience: Continuity of experience in psychotic and normal individuals. J. Nervous Mental Dis..

[35] Anselmetti S (2008). The brief assessment of cognition in schizophrenia. Normative data for the Italian population. Neurol. Sci..

[36] Castelnovo A, Aquilino D, Parabiaghi A, D’Agostino A (2020). Could CBT sustain long-term remission without antipsychotic medication in schizophrenia?. Schizophr. Res..

[37] Berry, R. B., Brooks, R., Gamaldo, C. E., Harding, S. M., Lloyd, R. M., Marcus, C. L., V. B. *The AASM Manual for the Scoring of Sleep and Associated Events: Rules, Terminology and Technical Specifications*. (2020).

[38] Castelnovo A (2016). Scalp and source power topography in sleepwalking and sleep terrors: A high-density EEG study. Sleep.

[39] Mensen A, Riedner B, Tononi G (2016). Optimizing detection and analysis of slow waves in sleep EEG. J. Neurosci. Methods.

[40] Nichols TE, Holmes AP (2002). Nonparametric permutation tests for functional neuroimaging: A primer with examples. Hum. Brain Mapp..

[41] Castelnovo A (2022). Sleep power topography in children with attention deficit hyperactivity disorder (ADHD). Children.

[42] Szeszko PR (2007). Clinical and neuropsychological correlates of white matter abnormalities in recent onset schizophrenia. Neuropsychopharmacology.

[43] Murphy M (2009). Source modeling sleep slow waves. Proc. Natl. Acad. Sci. USA.

[44] Piantoni G (2013). Individual differences in white matter diffusion affect sleep oscillations. J. Neurosci..

[45] Kurth S (2017). Traveling slow oscillations during sleep: A marker of brain connectivity in childhood. Sleep.

[46] Buchmann A (2011). Anatomical markers of sleep slow wave activity derived from structural magnetic resonance images. J. Sleep Res..

[47] Bernardi G (2021). Role of corpus callosum in sleep spindle synchronization and coupling with slow waves. Brain Commun..

[48] Hiatt JF, Floyd TC, Katz PH, Feinberg I (1985). Further evidence of abnormal non-rapid-eye-movement sleep in schizophrenia. Arch. Gen. Psychiatr..

[49] Ganguli R, Reynolds CF, Kupfer DJ (1987). Electroencephalographic sleep in young, never-medicated schizophrenics. A comparison with delusional and nondelusional depressives and with healthy controls. Arch. Gen. Psychiatry.

[50] Sekimoto M, Kato M, Watanabe T, Kajimura N, Takahashi K (2007). Reduced frontal asymmetry of delta waves during all-night sleep in schizophrenia. Schizophr. Bull..

[51] Sekimoto M, Kato M, Watanabe T, Kajimura N, Takahashi K (2011). Cortical regional differences of delta waves during all-night sleep in schizophrenia. Schizophr. Res..

[52] Keshavan MS (1998). Delta sleep deficits in schizophrenia: Evidence from automated analyses of sleep data. Arch. Gen. Psychiatry.

[53] Manoach DS (2014). Sleep spindle deficits in antipsychotic-naïve early course schizophrenia and in non-psychotic first-degree relatives. Front. Hum. Neurosci..

[54] Wamsley EJ (2012). Reduced sleep spindles and spindle coherence in schizophrenia: Mechanisms of impaired memory consolidation?. Biol. Psychiatry.

[55] Manoach DS (2010). Reduced overnight consolidation of procedural learning in chronic medicated schizophrenia is related to specific sleep stages. J. Psychiatr. Res..

[56] Göder R (2015). Impairment of sleep-related memory consolidation in schizophrenia: relevance of sleep spindles?. Sleep Med..

[57] Genzel L (2015). Medial prefrontal-hippocampal connectivity and motor memory consolidation in depression and schizophrenia. Biol. Psychiatry.

[58] Knott V, Labelle A, Jones B, Mahoney C (2001). Quantitative EEG in schizophrenia and in response to acute and chronic clozapine treatment. Schizophr. Res..

[59] Mong J, Cusumano D (2016). Sex differences in sleep: Impact of biological sex and sex steroids. Philos. Trans. R. Soc. B: Biol. Sci..

[60] Kaskie RE, Graziano B, Ferrarelli F (2019). Topographic deficits in sleep spindle density and duration point to frontal thalamo-cortical dysfunctions in first-episode psychosis. J. Psychiatr. Res..

